# Human but Not Laboratory Borna Disease Virus Inhibits Proliferation and Induces Apoptosis in Human Oligodendrocytes In Vitro

**DOI:** 10.1371/journal.pone.0066623

**Published:** 2013-06-21

**Authors:** Dan Li, Yang Lei, Jing Deng, Chanjuan Zhou, Yong Zhang, Wenjuan Li, Hua Huang, Shigang Cheng, Hongzhi Zhang, Liang Zhang, Rongzhong Huang, Xia Liu, Lihua Ma, Xiao Wang, Juan Li, Peng Xie

**Affiliations:** 1 Department of Pathology, Faculty of Basic Medicine, Chongqing Medical University, Chongqing, China; 2 Neuroscience Center, Key Laboratory of Neurobiology of Chongqing, Chongqing, China; 3 Department of Neurology, The First Affiliated Hospital, Chongqing Medical University, Chongqing, China; 4 Department of Neurology, The Chongqing Zhongshan Hospital, Chongqing, China; Massachusetts Eye & Ear Infirmary, Harvard Medical School, United States of America

## Abstract

Borna disease virus (BDV) is a neurotropic virus that produces neuropsychiatric dysfunction in a wide range of warm-blooded species. Several studies have associated BDV with human psychiatric illness, but the findings remain controversial. Although oligodendrocytes are a major glial component of brain white matter and play a pivotal role in neuronal cell function, BDV's effects on human oligodendrocytes have not been clarified. Here, the effects of two BDV strains, Hu-H1 (isolated from a bipolar patient) and Strain V (a laboratory strain), on the proliferation and apoptosis of human oligodendrocytes were investigated. Three experimental cell lines were constructed: Hu-H1-infected oligodendroglioma (Hu-H1) cells, Strain V-infected oligodendroglioma (Strain V) cells, and non-infected oligodendroglioma (control) cells. BDV infection was assayed by BDV nucleoprotein (p40) immunofluorescence, cell proliferation was assayed by Cell Counting Kit-8 (CCK8), and cell cycle phases and apoptosis were assayed by flow cytometry. Expressions of the apoptosis-related proteins Bax and Bcl-2 were measured by Western blotting. p40 expression was confirmed in Hu-H1 and Strain V on and after day three post-infection. Strain V cells showed significantly greater cellular proliferation than Hu-H1 cells on and after day three post-infection. In Hu-H1 cells, Bax and Bcl-2 expression were significantly increased and decreased, respectively, on and after day three post-infection. In contrast, in Strain V cells, Bax and Bcl-2 expression were significantly decreased and increased, respectively, on and after day three post-infection. In conclusion, Hu-H1 inhibits cellular proliferation and promotes apoptosis in human oligodendrocytes via Bax upregulation and Bcl-2 downregulation. In contrast, Strain V promotes cellular proliferation and inhibits apoptosis in human oligodendrocytes via Bax downregulation and Bcl-2 upregulation. The effects of the Hu-H1 strain (isolated from a bipolar patient) are opposite from those of Strain V (a laboratory strain), thereby providing a proof of authenticity for both.

## Introduction

It is well-known that many DNA viruses interact with the cell cycle machinery, as they are dependent on DNA synthetic enzymes for viral replication. Some viruses even induce cell apoptosis. In contrast, with the notable exceptions of human immunodeficiency virus (HIV) and respiratory syncytial virus (RSV), little is known about the interference of RNA viruses with cell cycle checkpoints and cell apoptosis [1]–[3].

Borna disease virus (BDV), a non-cytolytic single-stranded RNA virus, a member of the family *Bornaviridae* in the order *Mononegavirales* is highly neurotropic, infects a wide range of warm-blooded species, and in some cases, leads to T cell-mediated viral encephalitis or persistent infection of the central nervous system (CNS) [4]. A number of studies have associated BDV with human psychiatric illness, but the findings remain controversial [5]–[7]. However, BDV infection in neonatal rats (producing neonatal Borna disease [NBD]) does generate a persistent CNS infection with a range of neurodevelopmetal abnormalities and complex behavioral changes similar to those observed in autism, schizophrenia, and mood disorders (e.g., anxiety, abnormal playing behavior, deficits in spatial memory and learning). These behavioral and cognitive alterations may be attributed to virus induced neuronal loss or functional neuronal impairment, glial dysfunction, and/or modulation of the developmental fate of neural stem/progenitor cells (NSPCs) [8]–[10].

BDV infects neurons and glial cells, causing changes in nerve cell function and apoptosis of neurons and glial cells; however, the mechanism(s) of BDV infection in glial cells has been scarcely reported. Although oligodendrocytes are a major glial component of CNS white matter and play a pivotal role in neuronal cell function, human oligodendrocytes have not been well -studied in the context of BDV infection. Moreover, various cell types, rat strains, and viral strains have been shown to differentially affect BDV's influence on apoptotic activity [8], [11], [12]. Therefore, in this study, we investigated the proliferation and apoptosis of BDV Hu-H1 infected human oligodendroglioma (OL) cells and BDV Strain V-infected human OL cells to explore BDV's mechanism of action in human oligodendrocytes.

## Materials and Methods

### Viral Strains and Cell Lines

The virus strains (kindly provided by Prof. Hanns Ludwig of the Free University of Berlin, Germany) were a BDV Hu-H1 strain isolated from a bipolar patient's white blood cells [13],and a laboratory strain, Strain V, persistently infecting a human OL cell line, the biological and neurobiological properties of which had been outlined [14]. Each viral titer was approximately 2×10^5^ focus-forming units per milliliter (FFU/ml). The viral solution and OL cell lysate were obtained by freezing and thawing. All experiments were conducted in three cell lines: a BDV Hu-H1-infected human OL cell line (Hu-H1 cells), BDV Strain V-infected human OL cell line (Strain V cells), and an uninfected human OL cell line (control cells).

### Cell Culture

First, 10^3^ OL cells were plated in a volume of 100 µl into each well of two 96-well plates for a proliferation assay by Cell Counting Kit-8 (CCK8) (Beyotime, China). Meanwhile, 20 µl volumes of Hu-H1 and Strain V were added to each well of the Hu-H1 cells and Strain V cells, respectively. Thus, the multiplicity of infection (MOI) was approximately 4 FFU per OL cell (FFU/OL) for the Hu-H1 cells and Strain V cells.

To conduct the p40 immunofluorescence (IF) analysis, flow cytometric analysis for cell cycle and apoptosis, and the Western blot analysis for Bcl-2 and Bax, 10^5^ OL cells were plated in a volume of 1 ml into each well of twenty six-well plates, and then 200 µl volumes of Hu-H1 and Strain V were separately added to each well to form the Hu-H1 and Strain V cell lines. Thus, the multiplicity of infection (MOI) was approximately 0.4 FFU per OL cell (FFU/OL) for the Hu-H1 cells and Strain V cells.

The Hu-H1, Strain V, and control cells were all maintained in Dulbecco’s modified Eagle’s medium (DMEM) with high glucose (4.5 g/l) containing 1% penicillin,1% streptomycin, and 5% heat-inactivated fetal bovine serum (FBS). Cells were incubated at 37°C in a 5% CO2 incubator.After reaching 70–80% confluence, cells were washed with phosphate-buffered saline (PBS), harvested by 0.25% trypsinization, and resuspended in new medium. The cell density of the 96-well plate used for IF analysis was about 5×10^3^/ml, and about 5×10^5^/ml of cell density in a sixwell plate.

### IF Analysis

Hu-H1, Strain V, and control cells were grown to complete confluence, washed, trypsinized, and resuspended in PBS. Cells were smeared onto glass slides, dried, fixed in 4% paraformaldehyde solution for 30 min at room temperature, and then permeabilized in 0.4% Triton X-100 for 5 min, 3% hydrogen peroxide inactivated endogenous peroxidase for 30 min, and 5% bovine serum albumin (BSA) blocked non-specific antigen for 20 min. For BDV-specific protein detection, the slides were incubated with p40 polyclonal rabbit anti-BDV antibodies (100 µl, diluted 1∶50) in PBS for 12 h at 4°C, followed by fluorescein isothiocyanate (FITC)-conjugated goat anti-rabbit immunoglobulin G (IgG, 100 µl, diluted 1∶50) in PBS for 1 h at 37°C.

### Proliferation Assay

According to the manufacturer’s instructions, CCK8 (Beyotime, China) was used to measure the proliferation of Hu-H1, Strain V, and control cells. First, 10^3^ OL cells were plated in a volume of 100 µl into each well of two 96-well plates. All assays were performed in quadruplicate. After various incubation periods ranging from one to seven days, cells were incubated with the yellow CCK8 solution (10 µl) for approximately 2 h. After this incubation period, an orange soluble formazan product formed. The formazan product was spectrometrically quantified using an enzyme-linked immunosorbent assay reader (λ = 450nm), and growth curves were constructed.

### Flow Cytometric Analysis

#### Cell cycle analysis

Hu-H1, Strain V, and control cells were cultured without serum for 24 h to induce a G1 arrest. After the release from G1 arrest through addition of 5% serum to the medium, cells were harvested every 3 h over a 24-h period. Then, propidium iodide staining was performed to determine DNA content in the different cell cycle phases. For this staining, cells were washed, incubated with cold 70% ethanol overnight, and stained with 1 ml propidium iodide solution (50 µg propidium iodide/ml, 100 U of RNase A/ml, PBS) for 30 min. Cells were used for fluorimetric analysis. Flow-cytometric analysis for DNA content cannot distinguish between G2 and M. Therefore, the percentage of cells in G1, S, and G2/M phases was determined at different time points after release from G1 arrest.

#### Cell apotosis assay

According to the manufacturer’s instructions and van Engeland *et al.* [15], an annexinV FITC apoptosis detection kit (Beyotime, China) was used to measure apotosis in the Hu-H1, Strain V, and control cells. First, 10^5^ cells were plated in a volume of 1 ml into each well of a six-well plate. After various incubation periods of one, three, and five days, cells were trypsinized. Cells and the culture medium were collected. After 1000r centrifugation for 10 min, the supernatant was discarded. The cells were resuspended in 1 ml PBS, and transferred to the EP tube. Adding10 µl AnnexinV-FITC mixing on ice, in dark 15 min, cell apoptosis was detected by flow cytometry. Experiments were repeated in triplicate.

### Gel Electrophoresis and Western Blot Analysis

Gel electrophoresis and Western blot analysis were performed as previously described. For Western blot analysis, anti-Bax (sc-7480) and anti-Bcl-2(sc-7382) antibodies (both from Santa Cruz Biotechnology) were used. After incubation with species-specific per-oxidase-labeled secondary antibody, chemiluminescence was performed using BeyoECL Plus reagent. Toconfirm equal loading of the gel lanes, a Western blot analysis with anti-β-actin antibody (sc-58669, Santa Cruz Biotechnology) was used as an internal control.

### Statistical Analysis

SPSS 17.0 was used to analyze the experimental data. All experimental findings were mean ± standard deviations. The experimental cells were compared using ANOVA. A *p*-value of less than 0.05 was deemed to be statistically significant.

## Results

### p40 Protein Identification in BDV-Infected OL Cells

**Figure 1:**
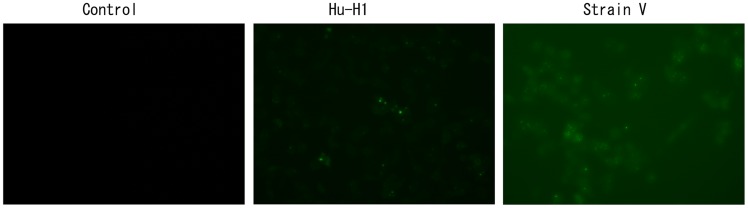
p40 expression on day three post-infection in Hu-H1 and Strain V cells.

BDV p40 protein was identified in the Hu-H1 and Strain V cells by IF on days one, three, and five post-infection. On day one post-infection, all three cell lines failed to reveal fluorescence expression. However, on days three and five post-infection, both the Hu-H1 and Strain V cells revealed fluorescent green focal points, but the control cells failed to reveal fluorescent expression. As BDV p40 protein is a nuclear protein, the visible green fluorescent protein is primarily expressed in the nucleus and confirms that Hu-H1 and Strain V cells were successfully infected with BDV (Figure 1).

### The Differing Effects of Hu-H1 and Strain V on OL Cell Proliferation

**Figure 2:**
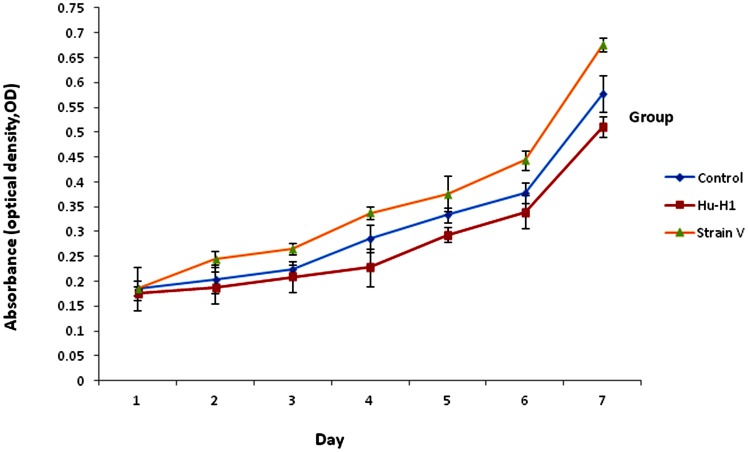
Cellular proliferation rates of Hu-H1, Strain V, and control cells.

The CCK8 assay (Table 1, Figure 2) revealed a gradual increase in OL cell proliferation from days one to seven in all three cell lines post-infection. On day one post-infection, there was no statistically significant difference in the proliferation of OL cells between the three cell lines by ANOVA (*p*>0.05).

**Table 1:** Optical densities of Hu-H1, Strain V, and control cells (mean±s, n = 3).

Time (d)	Group			*P*-value		
	Control	Hu-H1	Strain V	Control/Hu-H1# *p* = 0.000	Control/Strain V# *p = *0.000	Hu-H1/Strain V# *p* = 0.000
1	0.18535±0.0149265	0.17600±0.0438542	0.18615±0.0146561	0.671	0.931	0.645
2	0.20420±0.0159781	0.18820±0.0290052	0.24484±0.032460	0.311	*0.036	*0.020
3	0.22485±0.0110136	0.20925±0.0094340	0.26560±0.0302407	*0.044	*0.022	*0.012
4	0.286267±0.0136125	0.227867±0.0280303	0.337667±0.0386458	**0.003	*0.038	**0.001
5	0.33340±0.0351326	0.29320±0.0158335	0.37640±0.0149933	*0.012	*0.036	**0.000
6	0.377767±0.0195269	0.339367±0.0202287	0.443917±0.0331163	**0.008	**0.005	**0.000
7	0.577667±0.0138384	0.511167±0.0365205	0.675917±0.0215972	**0.005	**0.000	**0.000

# Repeated measures analysis of variance (ANOVA), *p*<0.01.

* Independent sample *t*-test, *p*<0.05.

** Independent sample *t*-test, *p*<0.01.

On day two post-infection, Strain V cells showed significantly greater OL cell proliferation than both the Hu-H1 cells (*p*<0.05, *p* = 0.020) and the control cells (*p*<0.05, *p* = 0.036); however, there was no statistically significant difference in OL cell proliferation between the Hu-H1 and control cells (*p*>0.05, *p* = 0.311).

On day three post-infection, the Strain V cells showed significantly greater OL cell proliferation than the control cells (*p*<0.05, *p* = 0.022), and the control group showed significantly greater OL cell proliferation than the Hu-H1 cells (*p*<0.05, *p* = 0.044). Logically, the Strain V cells also showed significantly greater OL cell proliferation than the Hu-H1 cells (*p*<0.05, *p* = 0.012).

The difference of the total results in the seven successive days between cell lines was statistically significant by ANOVA. Specifically, the Strain V cells showed significantly greater OL cell proliferation than the control cells (*p*<0.0001), and the control cells showed significantly greater OL cell proliferation than the Hu-H1 cells (*p*<0.0001). Logically, the Strain V cells also showed significantly greater OL cell proliferation than the Hu-H1 cells (*p*<0.0001).

### The Differing Effects of Hu-H1 and Strain V on the OL Cell Cycle

**Figure 3:**
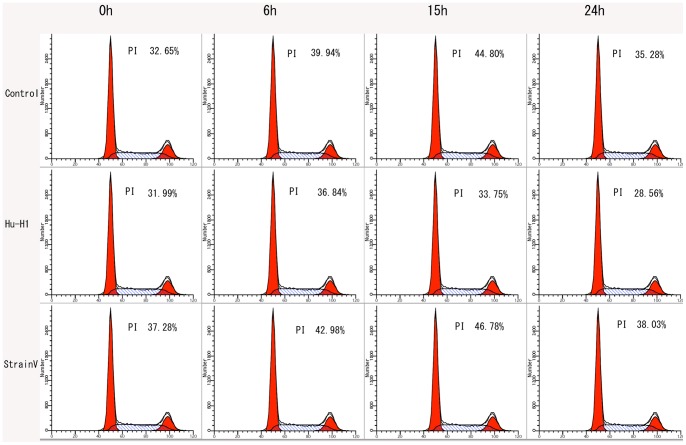
Cell cycle analysis of Hu-H1, Strain V, and control cells.

In the cell cycle, the primary phases of cell proliferation are the S and G2 phases. Thus, the cell proliferation index (PI), defined as the percentage of cells in S phase+G2/M phase, was applied to assess cell proliferation rates. Cell cycle phases were assayed by propidium staining and flow cytometry analysis (Figure 3).

**Figure 4:**
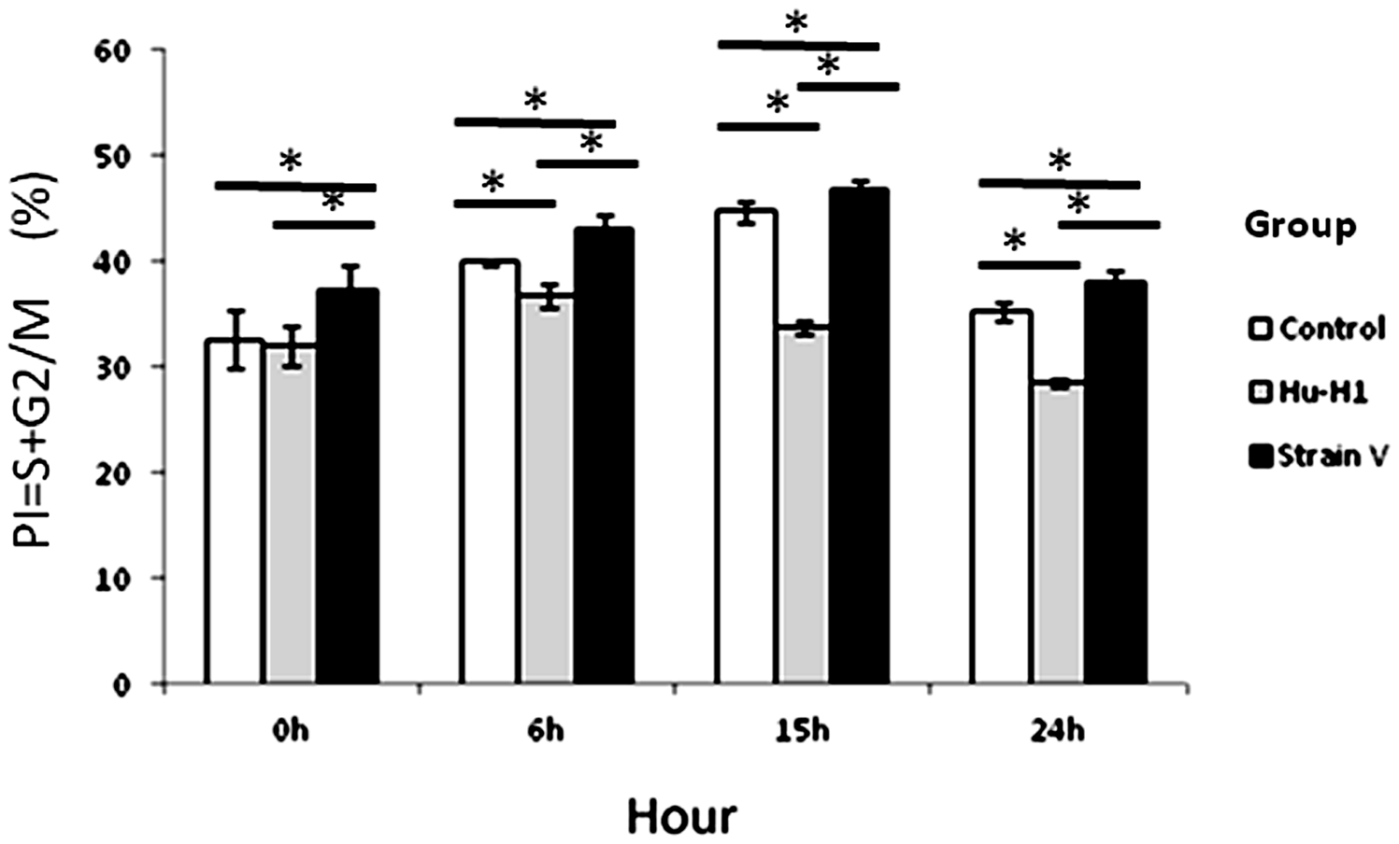
Proliferation indexes in Hu-H1, Strain V, and control cells. *p<0.05.

The Strain V cells showed a significantly lesser number of cells in the G0/G1 phase than both the control and Hu-H1 cells at 0 h post-serum release (*p<*0.05); however, there was no significant difference between the Hu-H1 and control cells in the G0/G1 phase at 0 h post-serum release (*p*>0.05). From 6 h post-serum release, the Hu-H1 cells showed a significantly greater number of cells in the G0/G1 phase (a lower proliferation index) than the control cells (*p*<0.05). From 0 h post-serum release, the control cells showed a significantly greater number of cells in the G0/G1 phase (a lower proliferation index) than the Strain V cells (*p*<0.05) (Table 2, Figure 4).

**Table 2:** Percentages of Hu-H1, Strain V, and control cells at various cell cycle phases (mean±s, n = 3).

Time (h)	Group	Cell cycle phase				P-value		
		G0/G1 (%)	G2/M (%)	S (%)	PI (%)	Control/Hu-H1	Control/Strain V	Hu-H1/Strain V
**0**	Control	67.35±2.68	4.00±0.53	28.65±2.70	32.65±2.68	*p*>0.05	*	*
	Hu-H1	68.01±2.96	3.97±0.32	28.02±1.88	31.99±1.86			
	Strain V	62.72±2.52	4.21±0.45	33.07±2.90	37.28±2.42			
**6**	Control	60.06±1.82	5.08±1.30	34.86±0.34	39.94±0.26	*	*	*
	Hu-H1	63.16±1.49	3.90±0.47	32.94±1.11	36.84±1.14			
	Strain V	57.02±1.53	5.53±1.25	37.45±0.96	42.98±1.34			
**15**	Control	55.20±0.66	7.69±1.32	37.10±1.10	44.80±0.99	*	*	*
	Hu-H1	66.25±0.98	4.18±1.35	29.57±0.69	33.75±0.57			
	Strain V	53.22±0.89	14.41±0.63	32.37±0.32	46.78±0.88			
**24**	Control	64.72±0.24	5.48±0.33	29.80±1.19	35.28±0.87	*	*	*
	Hu-H1	71.44±0.39	2.64±0.23	25.91±0.15	28.56±0.28			
	Strain V	61.97±1.08	6.36±0.56	31.66±0.91	38.03±1.08			

* statistical analysis of the cell proliferation index (PI), independent sample *t*-test, *p*<0.05.

### The Differing Effects of Hu-H1 and Strain V on OL Cell Apoptosis

**Figure 5:**
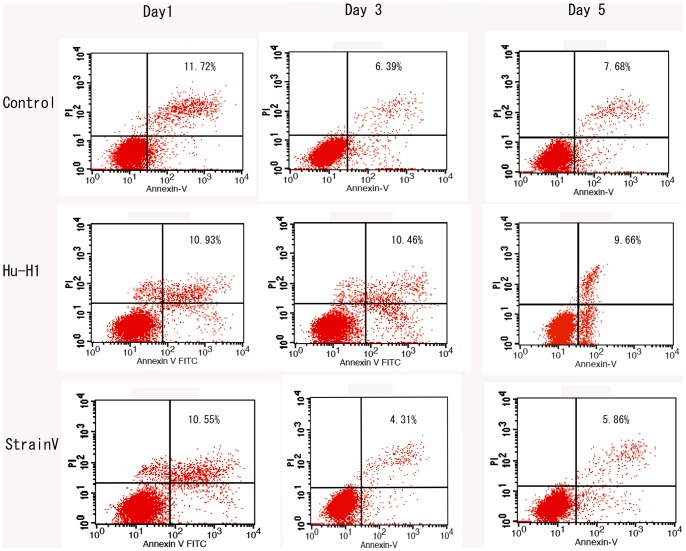
Apoptotic cell counts of Hu-H1, Strain V, and control cells.

**Figure 6:**
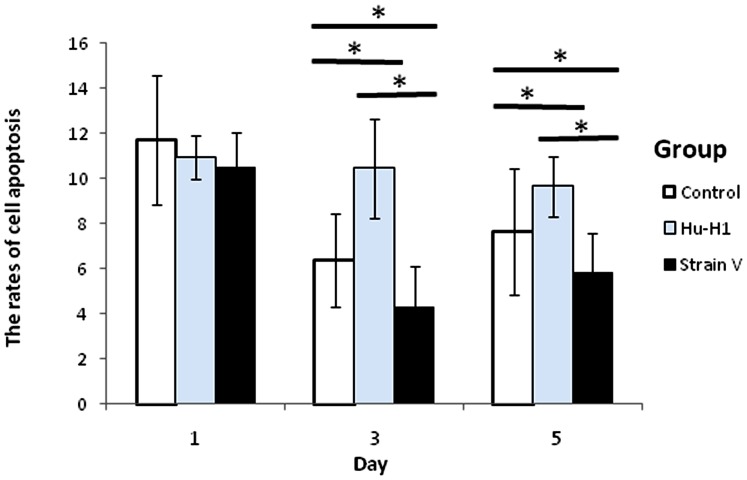
Rates of apoptosis in Hu-H1, Strain V, and control cells. *p<0.05.

With AnnexinV-FITC labeling, cell apoptosis was analyzed using flow cytometry on days one, three, and five post-infection (Figure 5, Table 3). There was no significant difference in the number of apoptotic cells between the three cell lines on day one post-infection. The Hu-H1 cells showed a significantly greater number of apoptotic cells than the control cells (*p*<0.05) on days three and day five post-infection, and the control cells showed a significantly greater number of apoptotic cells than the Strain V cells (*p*<0.05) on days three and five post-infection (Figure 6).

**Table 3:** Apoptotic cell counts at various time points (mean±s, n = 3).

Time (d)	Group			*P*-value		
	Control	Hu-H1	Strain V	Control/Hu-H1	Control/Strain V	Hu-H1/Strain V
1	11.72±2.85	0.93±2.06	0.55±2.81	*p*>0.05	*p*>0.05	*p*>0.05
3	6.39±0.971	10.46±2.19	9.66±1.79	*	*	*
5	7.68±1.491	4.31±1.34	5.86±1.74	*	*	*

* statistical analysis of apoptotic cell counts, independent sample *t*-test, *p*<0.05.

### The Differing Effects of Hu-H1 and Strain V on Apoptosis-Related Protein Expression

**Figure 7:**
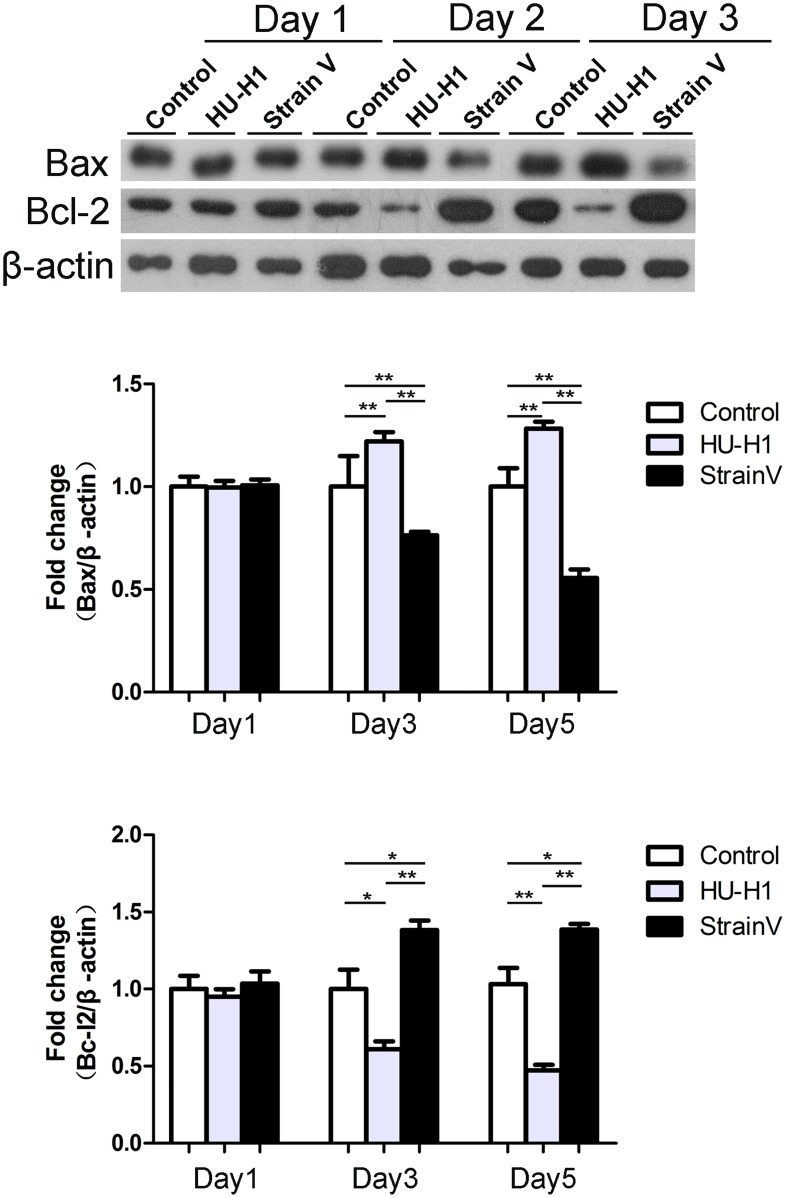
Bax and Bcl-2 expression in Hu-H1, Strain V, and control cells. *p<0.05 ; **p<0.05.

Through Western blot detection of the apoptosis-related proteins Bax and Bcl-2, the expression trend of these two proteins was found to be consistent with the flow cytometric results. On day one post-infection, the expression between the three cells did not significantly differ. On days three and five post-infection, pro-apoptotic Bax expression in the Hu-H1 cells was significantly greater than that in the control cells (*p*<0.01, *p* value were 0.0074 and 0.009 respectively), while anti-apoptotic Bcl-2 expression in the Hu-H1 cells was significantly lower than that in the control cells (*p*<0.05, *p* value were 0.047 and 0.007 respectively). In contrast, on days three and five post-infection, Bax expression in the Strain V cells was significantly lower than that of the control cells (p<0.01, *p* value were 0.008 and 0.0015 respectively), while Bcl-2 in the Strain V cells was significantly greater than that in the control cells (p<0.05, *p* value were 0.048 and 0.034 respectively). Logically, on days three and five post-infection, Bax expression in the Strain V cells was significantly lower than that of the Hu-H1 cells (p<0.001), while Bcl-2 in the Strain V cells was significantly greater than that in the Hu-H1 cells (p<0.001). (Figure 7).

## Discussion

BDV is a neurotropic virus that produces CNS dysfunction in a wide range of warm-blooded species from birds to non-human primates [6], [7], [16]. Its cellular entry follows a receptor mediated endocytotic pathway initiated by the viral glycoprotein G's recognition of an as-yet unidentified receptor at the cell surface [17].

BDV replicates in the nucleus of infected cells, and causes persistent infection and biomolecular changes in the cells to a certain degree by which manipulating the host cell’s metabolic network to support viral replication and proliferation [18]. Serologic and molecular epidemiologic evidence suggests that BDV is associated with certain neuropsychiatric disorders; however, this conclusion remains controversial [19]-[22]. Neonatally BDV-infected rats show persistent infection accompanied by neurodevelopmental abnormalities that may be attributed to virus-induced neuronal loss or functional neuronal impairment, glial dysfunction, and/or modulation of the developmental fate of NSPCs [8]–[10]. Ovanesov *et al.* found that persistent infection of neuronal cells leads to activation of non-infected astrocytes, which produces activation of microglia; this microglia activation in the absence of neuronal damage may represent the initial steps in the gradual neurodegeneration observed in brains of neonatally BDV-infected rats [23], [24].Therefore, the elucidation of BDV's mechanism of action in human brain cells is of great interest to both virologists and neuropsychiatric researchers alike.

### BDV Infection and Cellular Proliferation

On and after day three post-infection, both the Hu-H1 and Strain V cells revealed immunofluorescence, confirming that the Hu-H1 and Strain V cells were successfully infected with BDV (Figure 1). Consistently, Watanabe *et al.* also found that C6 cells showed positive immunofluorescence after day three post-infection [25]. The CCK8 assay showed that, on and after day three post-infection, the Strain V cells showed significantly greater OL cell proliferation than the Hu-H1 cells. Regulation of cellular proliferation occurs at two distinct cell cycle transitions: the G1 to S phase and the S to G2 phase. As cellular proliferation is initiated at the S phase, DNA content of the various cell cycle phases was assayed by flow cytometric analysis to investigate the effect of BDV infection on cellular proliferation; the PI was used as an indicator of cell proliferation [26]. From 0 h post-serum release, the Strain V cells showed a higher PI than the control cells (Table 2, Figure 4). From 6 h post-serum release, the Hu-H1 cells showed a lower PI than the control cells. These findings indicate that Strain V promotes OL cellular proliferation while Hu-H1 inhibits OL cellular proliferation. BDV's mechanism of action with respect to cellular proliferation remains unclear. The virus may directly interact with certain cell cycle regulatory proteins or may indirectly affect the expression of certain cytokines or growth factors that promote cellular proliferation. In cell systems using the laboratory strain He/80 the nucleoprotein (p40) was shown to interact with the CDC2-cyclin B1 complex resulting in G2 phase prolongation [27], whereas the phosphoprotein (p24) interacts with high mobility group box-1 (HMGB1) protein, an axonal growth factor that regulates transcription with RAG1, p53, and Hox, thereby interfering with cell signaling pathways and affecting nerve cells and brain development [28]. Additionally it was found that BDV CRP4 infection of PC12 cells activates the ERK1/2 pathway, hindering NGF-induced cell differentiation [29]. Further studies, however, are required to investigate BDV's interactions with cell cycle regulatory proteins and its effect on the expression of neurotrophic factors.

### Apoptosis

Apoptosis is a programmed cell death process that serves to remove abnormally proliferative cells, and BDV has been previously associated with apoptotic activity in brain cells. However, various cell types, rat species, and viral strains have been shown to differentially affect BDV's influence on apoptotic activity. For example, Williams *et al.* showed that BDV He/80 infection increases neuronal apoptosis and selective degeneration of granule cell neurons of the dentate gyrus (DG) in neonatal Lewis rats; mechanistically, increased expression of the pro-apoptotic proteins Fas and caspase-1 was accompanied by a decreased expression of the anti-apoptotic protein Bcl-2 [8]. In contrast, Poenisch *et al.,* also using the BDV He/80 strain, showed inhibition of apoptosis through increased anti-apoptotic X-protein activity in a rat astroglioma cell line (C6), and Wu *et al.* showed that granule cell neurons of the DG were unaffected in Sprague Dawley (SD) rats [11], [12].

Therefore, in this study, human OL cells were infected with two different BDV strains (Hu- H1 and Strain V), and then the number of apoptotic human OL cells was detected by flow cytometry. The two viral strains had disparate apoptotic effects; specifically, Hu-H1 promoted OL cell apoptosis, but Strain V inhibited OL cell apoptosis. In order to investigate the mechanism(s) of action behind these contrary effects, we focused on the expression of proteins in the apoptosis-related Bcl-2 family, which decide cell fate at the mitochondrial level [30]. Specifically, Western blotting was used to detect expression levels of the pro-apoptotic protein Bax and the anti-apoptotic protein Bcl-2. In the Hu-H1 cells, Bax and Bcl-2 expression were significantly increased and decreased, respectively, on days three and five post-infection. In contrast, in the Strain V cells, Bax and Bcl-2 expression were significantly decreased and increased, respectively, on days three and five post-infection. Therefore, Hu-H1 promotes cell apoptosis via Bax upregulation and Bcl-2 downregulation; in contrast, Strain V inhibits cell apoptosis via Bax downregulation and Bcl-2 upregulation.

### Conclusions

In conclusion, the findings on cell proliferation and cell apoptosis for the first time demonstrate that human wild-type BDV (Hu-H1) derived from a depressed patient and a laboratory adapted strain (Strain V) originally isolated from a diseased horse have significantly different biological properties in vitro (human OL cells). The latter one, however, had undergone several species crossings in animals and tissue culture cells during its passage history [14], and was shown to be genetically divergent from human strains [31]. These data not only are in accordance with similar studies using the adapted horse strain He/80 [8], [11], [23], [24], but underline the existence and difference of human- and animal BDV strains. Future efforts should focus on whether these effects are also present in vivo in the human host.
